# Emotional sounds in space: asymmetrical representation within early-stage auditory areas

**DOI:** 10.3389/fnins.2023.1164334

**Published:** 2023-05-19

**Authors:** Tiffany Grisendi, Stephanie Clarke, Sandra Da Costa

**Affiliations:** ^1^Service de Neuropsychologie et de Neuroréhabilitation, Centre Hospitalier Universitaire Vaudois (CHUV) and University of Lausanne, Lausanne, Switzerland; ^2^Centre d’Imagerie Biomédicale, Ecole Polytechnique Fédérale de Lausanne (EPFL), Lausanne, Switzerland

**Keywords:** human vocalizations, emotions, auditory belt areas, voice area, lateralization, 7T fMRI

## Abstract

Evidence from behavioral studies suggests that the spatial origin of sounds may influence the perception of emotional valence. Using 7T fMRI we have investigated the impact of the categories of sound (vocalizations; non-vocalizations), emotional valence (positive, neutral, negative) and spatial origin (left, center, right) on the encoding in early-stage auditory areas and in the voice area. The combination of these different characteristics resulted in a total of 18 conditions (2 categories x 3 valences x 3 lateralizations), which were presented in a pseudo-randomized order in blocks of 11 different sounds (of the same condition) in 12 distinct runs of 6 min. In addition, two localizers, i.e., tonotopy mapping; human vocalizations, were used to define regions of interest. A three-way repeated measure ANOVA on the BOLD responses revealed bilateral significant effects and interactions in the primary auditory cortex, the lateral early-stage auditory areas, and the voice area. Positive vocalizations presented on the left side yielded greater activity in the ipsilateral and contralateral primary auditory cortex than did neutral or negative vocalizations or any other stimuli at any of the three positions. Right, but not left area L3 responded more strongly to (i) positive vocalizations presented ipsi- or contralaterally than to neutral or negative vocalizations presented at the same positions; and (ii) to neutral than positive or negative non-vocalizations presented contralaterally. Furthermore, comparison with a previous study indicates that spatial cues may render emotional valence more salient within the early-stage auditory areas.

## Introduction

Three lines of evidence suggest that the spatial origin of sounds influences the perception of emotional valence. First, looming sounds tend to be perceived as more unpleasant, potent, arousing and intense than receding sounds (Bach et al., 2008, 2009; Tajadura-Jiménez et al., 2010b). Second, sounds were reported to be more arousing when presented behind than in front of a person and this effects was stronger for natural sounds, such as human or animal vocalizations, than tones (Tajadura-Jiménez et al., 2010a). Third, when presented in a dichotic paradigm emotional vocalizations were shown to yield asymmetrical behavioral scores. An early study used syllables without significance spoken in seven different emotional intonations. The performance in detecting one emotion, defined as target, was significantly better for stimuli presented to the left than the right ear (Erhan et al., 1998). A later study used four words, which differed in the initial consonant, and which were spoken in four different emotional intonations. The subjects attended either both ears or one of them at a time. Performance analysis revealed a significant left-ear advantage for identifying the emotion (Jäncke et al., 2001). The behavioral results of either study were interpreted in terms of right hemispheric competence for emotional processing (e.g., Gadea et al., 2011), a concept which has been established in activation studies using non-lateralized stimuli (Frühholz and Grandjean, 2013; Frühholz et al., 2016). The alternative interpretation, that the emotional perception may be modulated by the lateralization of the sound, as it is for looming vs. receding sounds (Bach et al., 2008, 2009; Tajadura-Jiménez et al., 2010b), has not been considered.

The encoding of the auditory space is believed to be partially independent of the encoding of sound meaning. A series of seminal studies lead to the formulation of the dual-stream model of auditory processing, which posits partially independent encoding of sound meaning along the anterior temporal convexity and that of sound position on the parietal convexity. The functional independence of the two pathways has been documented in patient studies, where lesions limited to the ventral stream impaired sound recognition but not localization and conversely lesions limited to the dorsal stream impaired sound localization but not recognition (Clarke et al., 2000, 2002; Rey et al., 2007).

Recent evidence indicates that the combined encoding of sound object identity and location involves a separate, third processing stream, referred to also as the lateral pathway (Clarke and Geiser, 2015). Its initial demonstration relied on repetition priming paradigms; neural populations, which encoded the combined representation, displayed repetition enhancement when an object changed position and repetition suppression when it did not, both in EEG (Bourquin et al., 2013) and in 7T fMRI experiments (Da Costa et al., 2018). The latter identified several early-stage auditory areas on the supratemporal plane which participate in the combined encoding of sound object identity and position. The position-linked representation of sound objects, as supported by the lateral auditory pathway, is likely to contribute to auditory streaming, where spatial cues play an important role in the very early processing stages (Eramudugolla et al., 2008). The functional independence of the lateral and dorsal auditory pathways, has been demonstrated in patient studies, where the implicit use of auditory spatial cues was preserved for the segregation of sound objects, despite severe sound localization deficits, including cortical spatial deafness (Thiran and Clarke, 2003; Duffour-Nikolov et al., 2012; Tissieres et al., 2019).

The early-stage primary and non-primary auditory areas are located on the supratemporal plane and constitute first steps of cortical processing; several of them were defined by anatomical, histological and/or functional markers in post-mortem studies and by functional criteria (Clarke and Morosan, 2012). The primary auditory cortex is roughly co-extensive with Heschl’s gyrus (Zilles et al., 1988; Rademacher et al., 2001) and consists of two orderly tonotopic representations (Formisano et al., 2003; Da Costa et al., 2011, 2014; Moerel et al., 2014). The surrounding plana polare and temporale comprise several non-primary auditory areas, which were characterized on the basis of histological criteria (Rivier and Clarke, 1997; Clarke and Rivier, 1998; Hackett et al., 2001; Wallace et al., 2002; Chiry et al., 2003). Their Talairach coordinates were used in activation studies (Viceic et al., 2006; van der Zwaag et al., 2011; Besle et al., 2019), in addition to the identification of the primary auditory cortex by means of tonotopic mapping (Da Costa et al., 2011, 2015, 2018).

Human vocalizations constitute emotionally highly potent stimuli. They are processed in a dedicated region on the superior temporal gyrus, the voice area (VA), which is defined by its stronger response to human than animal vocalizations (Belin et al., 2000). The encoding of vocalizations within VA is modulated by emotional valence, as demonstrated in a series of seminal studies (Belin et al., 2002; Grandjean et al., 2005; Ethofer et al., 2006, 2008, 2009, 2012; Beaucousin et al., 2007; Obleser et al., 2007, 2008; Bestelmeyer et al., 2017). In addition to VA, the emotional valence of vocalizations impacts also the activity on Heschl’s gyrus and the antero-lateral part of the planum temporale (Wildgruber et al., 2005; Leitman et al., 2010; Ethofer et al., 2012; Arnal et al., 2015; Lavan et al., 2017). The relatively low spatial resolution used in these studies did not allow to analyze separately neural activity within VA and within individual auditory areas. This has been done in a recent 7T fMRI study, which used human vocalizations and non-vocalizations with positive, neutral or negative valence (Grisendi et al., 2019). Several early-stage auditory areas yielded stronger responses to non-verbal vocalizations and/or were modulated by emotional valence. In contrast, in VA emotional valence selectively modulated the responses to human vocalizations but not to non-vocalizations.

Emotional valence appears to impact differently the processing within the ventral and dorsal auditory streams. An fMRI study investigated neural activity elicited by environmental sounds, which consisted to 75% of human vocalizations with positive, neutral or negative valence and were presented at one of two left or two right positions; the authors report a main effect of position; driven by a stronger activity to contralateral stimuli; bilaterally in a temporo-parietal region. A main effect of emotion, driven by stronger activity to emotional than neutral stimuli, was present bilaterally in an antero-superior temporal region. A significant interaction between position and emotional valence, driven by stronger response to contralateral positive stimuli, was found in the right auditory cortex (Kryklywy et al., 2013). In a follow-up study (Kryklywy et al., 2018) the data were re-analyzed with multi-voxel pattern analysis, which revealed overlapping representations of spatial and emotional attributes within the posterior part of the supratemporal plane.

In summary, human vocalizations strongly convey emotional valence, with a major involvement of VA and of the postero-lateral part of the planum temporale (Wildgruber et al., 2005; Leitman et al., 2010; Ethofer et al., 2012; Arnal et al., 2015; Lavan et al., 2017). The perceived emotional valence of sounds, including vocalizations, is modulated by spatial attributes as demonstrated for looming sounds (Bach et al., 2008, 2009; Tajadura-Jiménez et al., 2010b). A likely candidate for the interaction between emotional valence and spatial attributes of sounds is the planum temporale (Kryklywy et al., 2018). It is currently unclear whether other spatial attributes, such as left vs. right locations (and not simply left vs. right ear), modulate emotional perception and its encoding as well, and whether human vocalizations vs. other environmental sounds differ in this respect. We have addressed these issues and hypothetized that specific early-stage auditory areas and/or VA may display one or several of the following characteristics:

- The encoding of emotional vocalizations, but not of other emotional sounds, is more strongly modulated by their position than that of neutral vocalizations or non-vocalizations;
- The encoding of emotional valence, independently whether the stimuli are human vocalizations or other environmental sounds, is modulated by the spatial origin of the sound;
- The spatial origin of the sound has differential effect on the encoding of vocalizations vs. other environmental sounds.

Furthermore, we expected to find spatial, emotional and vocalization selectivity, as reported in previous studies (Belin et al., 2002; Grandjean et al., 2005; Wildgruber et al., 2005; Ethofer et al., 2006, 2008, 2009, 2012; Beaucousin et al., 2007; Obleser et al., 2007, 2008; Leitman et al., 2010; Kryklywy et al., 2013; Arnal et al., 2015; Bestelmeyer et al., 2017; Lavan et al., 2017; Da Costa et al., 2018; Grisendi et al., 2019). To test the three hypotheses, we have made use of the high spatial resolution of ultra-high field fMRI at 7T to investigate the representation of human vocalizations vs. other environmental sounds, and their modulation by emotional valence and/or by their position within early-stage auditory areas and VA.

## Materials and methods

### Participants

Thirteen subjects (9 female, 11 right-handed, mean age 26.54 ± 4.31 years) participated in this study. All subjects were native speakers of French, without musical training. None reported history of neurological or psychiatric illness or hearing deficits and all had hearing thresholds within normal limits. Prior to the imaging session, each subject completed six questionnaires on their health status, handedness [Edinburgh Handedness Inventory, (Oldfield, 1971)], anxiety and depression state [Hospital Anxiety and Depression, HAD, scale; (Zigmond and Snaith, 1983)], personality traits [Big-Five Inventory, (Courtois et al., 2018)], and a musical aptitude questionnaire developed in the lab. These questionnaires revealed no significant differences in personality traits nor in the presence of mood disorders between our subjects and normal population. The experimental procedures were approved by the Ethics Committee of the Canton de Vaud; all subjects gave written, informed consent.

### Experimental design and statistical analysis

The experimental design consisted of two fMRI sessions (~55–60 min each) during which auditory stimuli were presented while the subjects listened passively to the stimuli with eyes closed. In total, each subject performed two runs of tonotopy mappings, one run of voice localizer, and 12 runs of “emotions&space” runs. Each of the latter consisted of 20s of silent rest (with no auditory stimuli except the scanner noise), followed by nine 36 s-blocks of 11 sounds of the same condition (22 s sounds and 14 s of silent rest), and again 20 s of silent rest. Each block was composed of 11 different sounds from the same category (human vocalizations or other environmental sounds), all of which had the same emotional valence (positive, neutral or negative) and the same lateralization (left, center, right). Finally, blocks and their sequence order were pseudo-randomized within runs and across subjects.

Sounds (16 bits, stereo, sampling rate of 41 kHz) presented binaurally at 80 ± 8 dB SPL via MRI-compatible headphones (SensiMetrics S14, SensiMetrics, United States), with a prior filtering with the SensiMetrics filters to obtain a flat frequency transmission, using MATLAB (R2015b, The MathWorks, Inc., Natick, Massachusetts, United States) and the Psychophysics Toolbox^1^. The auditory stimuli were the same as the battery used in previous studies (Aeschlimann et al., 2008; Grisendi et al., 2019), the total 66 different emotional sound files were 2 s-long and were equally distributed in the six categories: Human Vocalizations Positive (HVP; e.g., baby or adult laughing; erotic vocalizations by man or woman), Human Vocalizations Neutral (HV0; vowels or consonant-vowels without significance), Human Vocalizations Negative (HVN; e.g., frightened scream; vomiting; brawl), Non-Vocalizations Positive (NVP; e.g., applause; opening beer can and pouring into a glass; river), Non-Vocalizations Neutral (NV0; e.g., running car engine; wind blowing; train), and Non-Vocalizations Negative (NVN; e.g., ticking and exploding bomb; tire skids; breaking glass). Sounds were lateralized by creating artificially a temporal shift of 0.3 s between the left and right channel (corresponding to ~60°), using the software Audacity (Audacity Team^2^), and were either perceived as presented on the left, the center or the right auditory space. Thus, the combination of all the different characteristics resulted in a total of 18 conditions (2 Categories x 3 Valences x 3 Lateralizations).

As previously, using a specific software, PRAAT^3^, and MATLAB scripts, the sound acoustic characteristics (spectrograms, mean fundamental frequency, mean intensity, harmonics to noise ratio, power, center of gravity, mean Wiener entropy and spectral structure variation) were controlled for each category: first, the significant differences between the mean spectrogram of pairs of sounds of different categories were maintained <1% to avoid bias toward a specific category (as in De Meo et al., 2015); second, all the sounds characteristics were tested with a two-way repeated measures ANOVA with the factors Category (Human-Vocalizations, Non-Vocalizations) x Valence (Positive, Neutral, Negative) to compare the effect of each acoustic feature on the sound categories. As already reported in our previous study (Grisendi et al., 2019), the analysis on mean Wiener entropy showed a main effect of Category [*F*(1,64) = 18.68, *p* = 0.0015], a main effect of Valence [*F*(2,63) = 21.14, *p* = 1.17E-5] and an interaction Category x Valence [*F*(2,63) = 8.28, *p* = 0.002]; while the same analysis on the center of gravity revealed a main effect of Valence [*F*(2,63) = 10.51, *p* = 0.0007]. The analysis of the harmonics-to-noise ratios highlighted a main effect of Category [*F*(1,64) = 134.23, *p* = 4.06E-7], a main effect of Valence [*F*(2,63) = 69,61, *p* = 9.78E-10] and an interaction of Category x Valence [*F*(2,63) = 17.91, *p* = 3.48E-5], and these of the power showed an interaction of Category x Valence on the mean intensity [*F*(2,63) = 12.47, *p* = 0.0003] and on the power [*F*(2,63) = 14.77, *p* = 0.0001].

### Regions of interest definition

The subdivision of the early-stage auditory areas was carried out in individual subjects as described previously (Da Costa et al., 2015, 2018). The subjects listened to two runs (one ascending and one descending) of a tonotopic mapping paradigm, which consisted of progressions of 2 s-bursts of pure tones (14 frequencies, between 88 and 8,000 Hz, in half octave steps) presented in 12 identical cycles of 28 s followed by a 12-s silent pause for a total duration of 8 min (as in previous studies Da Costa et al., 2011, 2013, 2015, 2018). Then, briefly, based on the resulting individual frequency reversals and anatomical landmarks, each early-stage auditory area was localized and defined in each subject as the primary auditory cortex, A1 and R, as well as the lateral (L1, L2, L3, L4) and medial non-primary areas (M1, M2, M3, M4). The coordinates of these regions were in accordance with previously published values (Table 1; Viceic et al., 2006; van der Zwaag et al., 2011; Da Costa et al., 2015, 2018).

**Table 1:** Mean MNI coordinates (center of gravity) of all ROIs.

ROI	X		Y		Z	
	Mean	STD	Mean	STD	Mean	STD
*Left hemisphere*						
A1	−34.81	30.29	−28.11	5.64	9.15	3.93
R	−42.84	4.84	−21.79	5.11	7.50	4.37
L1	−57.25	5.74	−37.57	7.71	18.06	8.46
L2	−58.14	5.55	−21.17	6.22	6.76	5.49
L3	−52.89	5.41	−9.08	6.75	0.60	4.67
L4	−45.23	4.38	−4.18	11.06	−11.17	7.63
M1	−46.51	5.74	−38.74	4.57	23.72	7.95
M2	−36.03	2.71	−33.44	2.85	17.74	3.36
M3	−33.14	2.80	−29.26	2.50	17.48	3.25
M4	−35.80	3.17	−14.80	9.49	−2.84	11.85
VA	−55.50	6.47	−33.46	10.53	6.08	5.62
*Right hemisphere*						
A1	49.54	5.19	−23.74	5.08	10.60	3.49
R	45.49	4.65	−17.56	4.88	6.73	4.83
L1	60.92	5.04	−30.13	4.67	21.69	9.94
L2	62.40	4.10	−18.00	7.27	7.07	4.57
L3	55.99	5.43	−4.77	6.81	−0.24	4.68
L4	46.90	4.63	−0.42	10.06	−11.67	6.99
M1	48.99	6.26	−31.56	3.64	26.70	8.41
M2	38.04	3.49	−29.90	3.19	18.33	3.83
M3	35.14	3.12	−26.33	3.43	16.28	4.06
M4	34.95	2.92	−10.74	10.96	−3.30	10.32
VA	48.79	7.60	−31.39	7.37	5.46	4.98

STD, standard deviation.

Finally, the position of VA was defined using a specific voice localizer (Belin et al., 2002; Pernet et al., 2015). Briefly, human vocalizations (vowels, words, syllables laughs, sighs, cries, coughs, etc.) and environmental sounds (falls, wind, animals sounds, etc.) were presented in a 10-min run, which consisted of forty 20s-long blocks (with 8 s of sounds followed by a silent pause of 12 s). This localizer was developed to easily and consistently identify the individual voice area along the lateral side of temporal plane, by displaying the results of the general linear model (GLM) contrast Human vocalizations vs. Environmental sounds. In this study, the same approach was used in BrainVoyager (BrainVoyager 20.6 for Windows, Brain Innovation, Maastricht, Netherlands). After initial preprocessing, the functional run was first aligned with the subject anatomical, and analyzed with a general linear model using a boxcar design for the two conditions. Second, the results of the contrast Human vocalization vs. Environmental sounds was projects on the individual 3D volume rendering with a *p* value of *p* < 0.005 (uncorrected) in order to cover the same extend in each subject. Finally, the activated region within the bilateral lateral borders of the STS/STG was manually selected as a patch of interest using the manual drawing tools from BrainVoyager and projected back into the MNI space and saved as the individual region of interest. The coordinates of the VA were also in accordance with those of previous studies (Belin et al., 2002; Pernet et al., 2015).

### Imaging parameters and data analysis

Brain imaging was acquired on a 7-Tesla MRI scanner (Siemens MAGNETOM scanner, Siemens Medical Solutions, Germany) with an 32-channel head RF-coil (Nova Medical Inc., MA, United States). Functional datasets were obtained with a 2D-EPI sinusoidal simultaneous multi-slice sequence (1.5 × 1.5 mm in-plane resolution, slice thickness = 1.5 mm, TR = 2000 ms, TE = 23 ms, flip angle = 90°, slice gap = 0 mm, matrix size = 146 × 146, field of view = 222 × 222, with 40 oblique slices covering the superior temporal plane). T1-weigthed 3D structural images were obtained with a MP2RAGE sequence [resolution = 0.6 × 0.6 × 0.6 mm^3^, TR = 6,000 ms, TE = 4.94 ms, TI1/TI2 = 800/2700 ms, flip angle 1/flip angle 2 = 7/5, slice gap = 0 mm, matrix size = 320 × 320, field of view = 192 × 192 (Marques et al., 2010)]. Finally, the physiological noise (respiration and heart beat) was recorded during the experiment using a plethysmograph and respiratory belt provided from the MRI scanner vendor.

The data was processed with BrainVoyager with the following steps: scan time correction (except for tonotopic mappings runs), temporal filtering, motion correction, segmentation and normalization into the MNI space. Individual frequency preferences were extracted with a linear cross-correlation analysis, resulting correlation maps were averaged together (ascending and descending correlation map) to define the best frequency value for each voxel in the volumetric space, and then the average map was projected onto the cortical surface meshes for the ROIs definition (Da Costa et al., 2011, 2013, 2015, 2018). For the VA localizer and the emotion&space runs, a random effects (RFX) analysis was performed at the group level, with movement and respiration parameters as regressor, and then we tested for the contrast ‘Sounds vs. Silence’ with an FDR correction at *q* < 0.05 (*p* < 0.05). The GLM results for the VA localizer was used to outline the VA in the left and in the right hemisphere of each individual brain, while the GLM results for the emotion&space runs were used to verify that our ROIs were activated by the paradigm. The scope of this paper was to evaluate the effects of spatial origin on the encoding of emotional sounds, therefore the remaining analysis focused on the BOLD responses extracted from all the ROIs.

Functional individual BOLD time courses were processed as the following: first, they were extracted using BrainVoyager, imported into MATLAB. Second, they were normalized by their own mean signal, and divided according to their condition. Third, they were averaged spatially (across all voxels within each ROI), temporally (over blocks and runs), and across the 13 subjects. The resulting time course consisted of 18 time points for each ROI and condition. Finally, these time courses were analyzed with a time-point-by-time-point Three-Way repeated measure ANOVA, two Category (Human-Vocalizations, Non-Vocalizations) x 3 Valence (Positive, Neutral, Negative) x 3 Lateralization (Left, Center, Right) according to Da Costa et al. (2015, 2018) and Grisendi et al. (2019). This three-way ANOVA was further decomposed for each vocalization category onto a two-way repeated measure ANOVA, 3 Valence (Positive, Neutral, Negative) x 3 Lateralization (Left, Center, Right). For each ANOVA, and each pair of condition, *post hoc* time-point-by-time-point paired *t*-tests were performed to evaluate the causality of the effects. Finally, results were restricted temporally by only considering at least three consecutive time points with significant *p*-values lower or equal to 0.05.

### Physiological noise processing

Heartbeat and respiration recordings were processed with an open-source toolbox for Matlab, TAPAS PhysIO (Kasper et al., 2017). The cardiac rates were further analyzed with the same pipeline as the BOLD responses to obtain a pulse time course for each condition, while the respiration rates were used within the GLM model as motion regressor. The effect of space and emotional contents of the sounds on the individual cardiac rhythm was evaluated by computing the heart rate variability as reported in previous studies by others (Goedhart et al., 2007) and by us (Grisendi et al., 2019).

## Results

**Figure 1:**
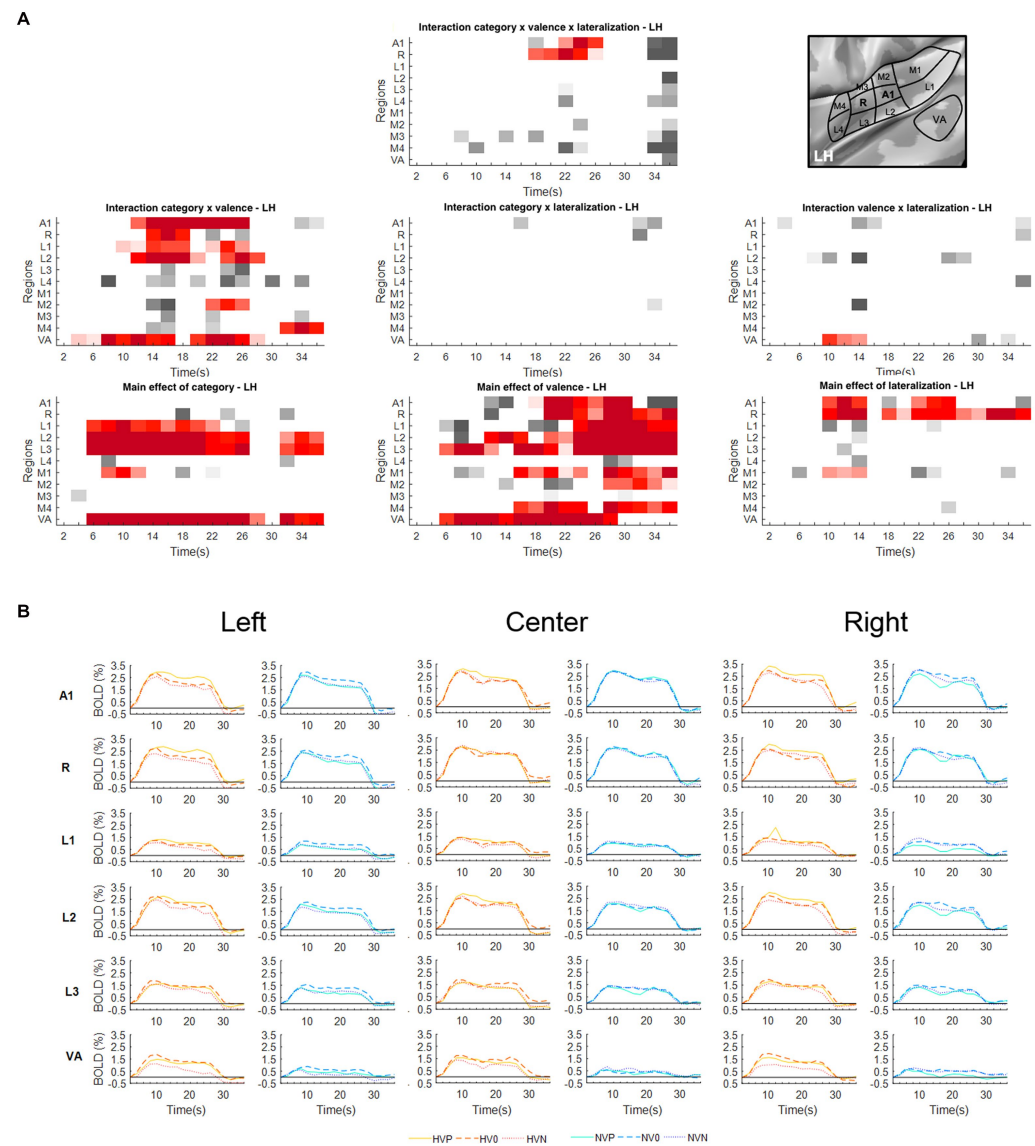
Activations elicited in the left hemisphere. **(A)** Statistical analysis of the BOLD signal by means of a two-way ANOVA with factors Vocalization (vocalizations, non-vocalizations) x Valence (positive, neutral, negative) x Lateralization (left, center, right). The ROIs, i.e., early-stage auditory areas and VA, are represented on the *y*-axis, the time points on the *x*-axis; red indicates a value of *p* lower or equal to 0.05 for at least three consecutive time points, gray a value of *p* lower or equal to 0.05 for isolated time-points. LH, left hemisphere. **(B)** BOLD time courses for selected early-stage areas and VA, presented on the left, at the center or on the right. Human vocalization categories are depicted in orange [HVP (solid line), HV0 (dashed line), HVN (dotted line)] non-vocalization categories in blue [NVP (solid line), NV0 (dashed line), NVN (dotted line)]. Full line denotes positive, interrupted line neutral and dotted line negative valence. The inset in top right corner shows the location of early-stage auditory areas on unfolded view of Heschls gyrus, its delimiting sulci as well as the anterior part of the planum temporale and the posterior part of the planum polare (gyri are in light, sulci in dark gray; medial is up, anterior to the right).

**Figure 2:**
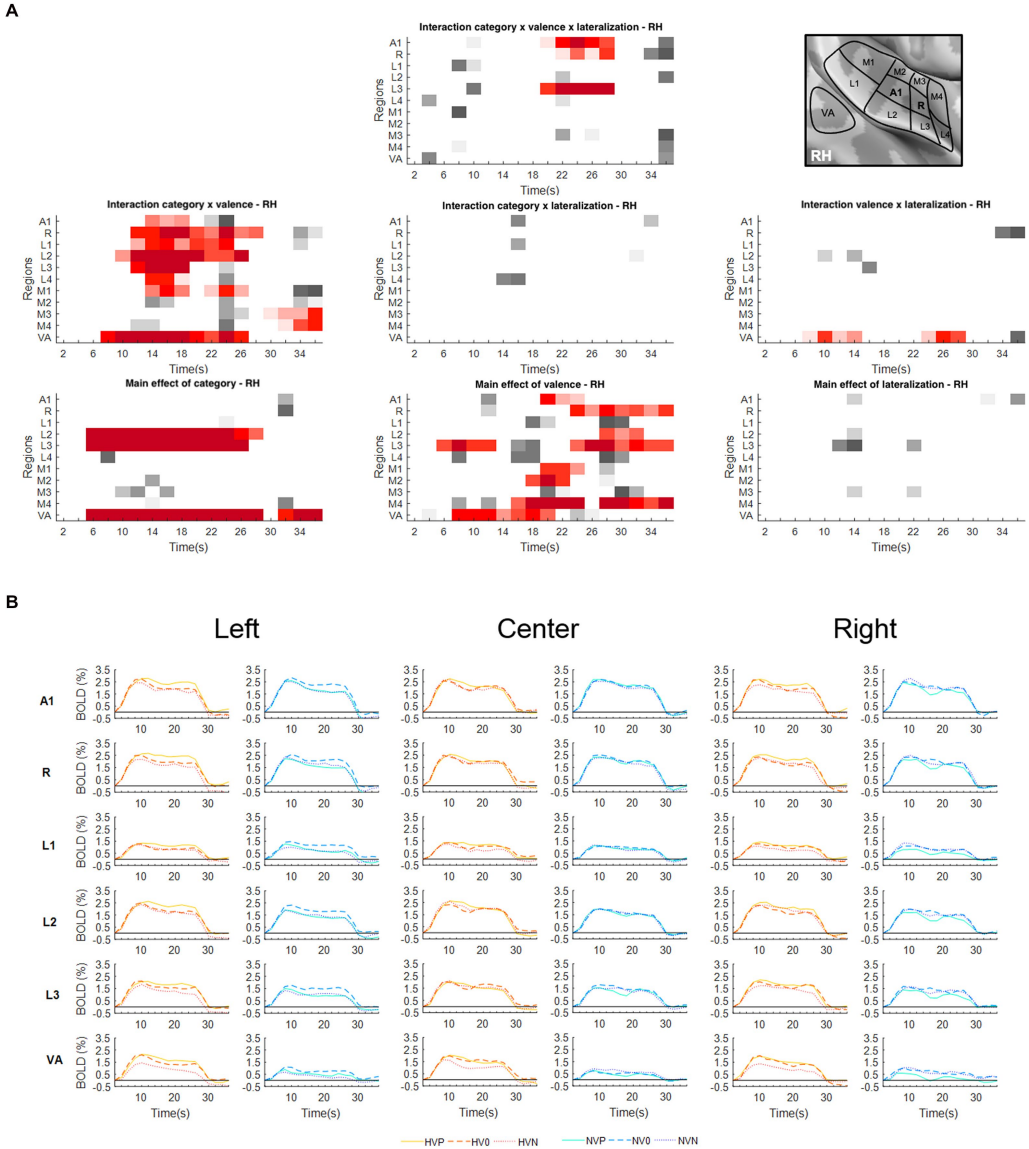
Activations elicited in the right hemisphere. **(A)** Statistical analysis of the BOLD signal by means of a two-way ANOVA with factors Vocalization (vocalizations, non-vocalizations) x Valence (positive, neutral, negative) x Lateralization (left, center, right). The ROIs, i.e., early-stage auditory areas and VA, are represented on the *y*-axis, the time points on the *x*-axis; red indicates a value of *p* lower or equal to 0.05 for at least three consecutive time points, gray a value of *p* lower or equal to 0.05 for isolated time-points. RH, right hemisphere. **(B)** BOLD time courses for selected early-stage areas and VA, presented on the left, at the center or on the right. Human vocalization categories are depicted in orange [HVP (solid line), HV0 (dashed line), HVN (dotted line)] non-vocalization categories in blue [NVP (solid line), NV0 (dashed line), NVN (dotted line)]. Full line denotes positive, interrupted line neutral and dotted line negative valence. The inset in top right corner shows the location of early-stage auditory areas on unfolded view of Heschls gyrus, its delimiting sulci as well as the anterior part of the planum temporale and the posterior part of the planum polare (gyri are in light, sulci in dark gray; medial is up, anterior to the left).

To explore to what extent emotional valence and/or position modulate the encoding of vocalizations vs. non-vocalizations within specific ROIs, we have analyzed the BOLD responses within each area with a three-way repeated measure ANOVA with factors Valence (Positive, Neutral, Negative), Lateralization (Left, Center, Right) and Category (Human-Vocalizations, Non-Vocalizations). The significance of main effects and interactions within individual early-stage auditory areas and within VA (Figures 1, 2) provided answers for the three hypotheses we set out to test.

The encoding of emotional vocalizations is more strongly modulated by their position than that of neutral vocalizations or non-vocalizations (hypothesis 1).

**Figure 3:**
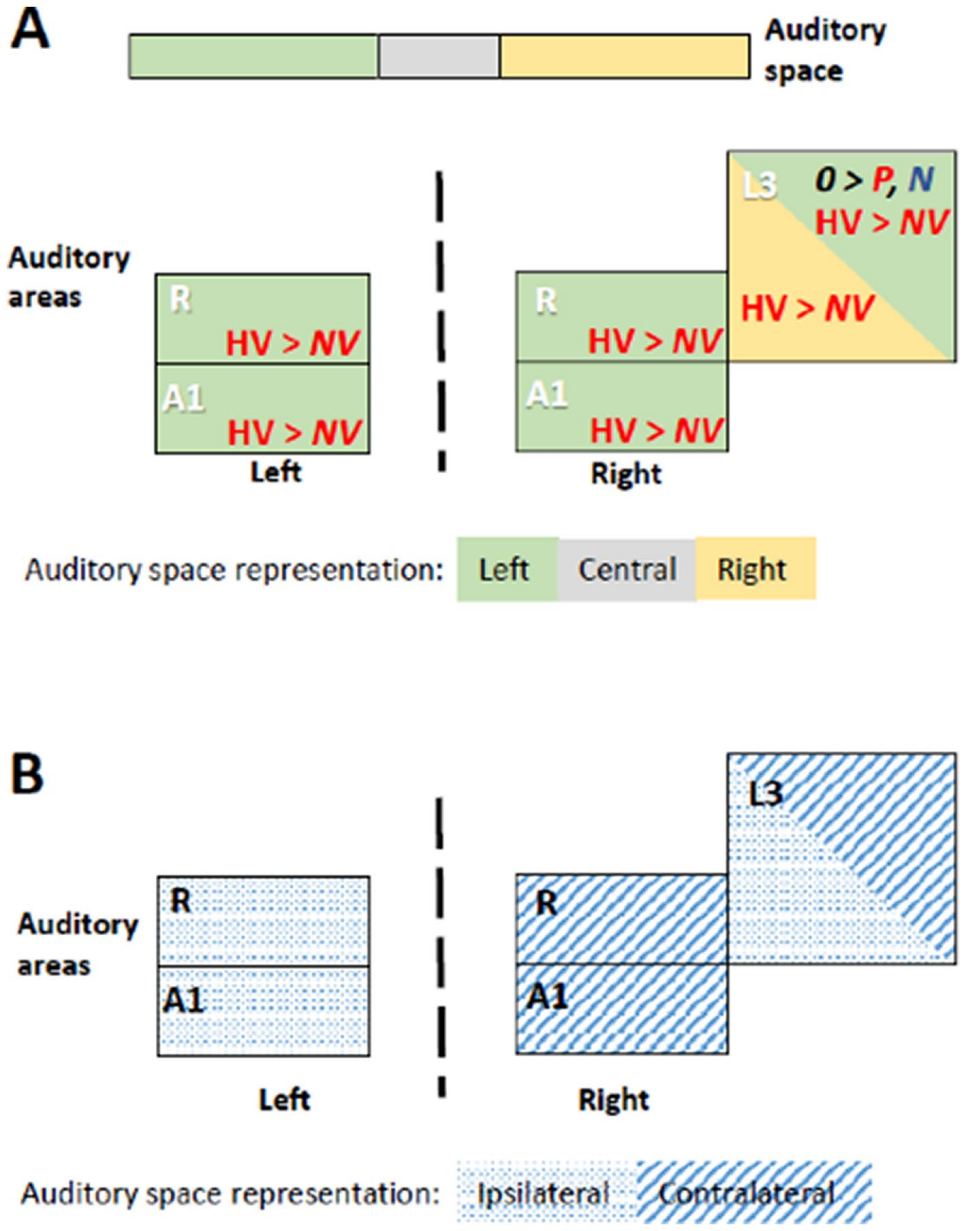
Summary of significant effects demonstrating differential processing of category, valence and space within early-stage auditory areas. Within the timeframe of significant triple interaction Category x Valence x Space, ensuing double dissociations and main effects were analyzed (Table 2), revealing significant effects for subgroups of stimuli. **(A)** Significant effect occurred when stimuli were presented at specific locations. When presented within the left space, positive human vocalizations yielded greater responses than neutral or negative ones in right and in left areas A1 and R. They yielded also greater responses in right L3, when presented on the left or on the right side. In addition, in right L3 neutral non-vocalizations yielded greater responses than positive or negative ones, when presented on the left. Green denotes left, gray central, and yellow right auditory space. Within auditory areas, the same colors denote the part of space for which the effect was significant. Red ink denotes positive, blue negative and black neutral valence. Italic font highlight non-vocalization, upright human vocalizations. **(B)** Left and right primary auditory areas A1 and R differed in their preference for auditory space; on the left side they responded differentially for stimuli presented ipsilaterally, on the right side contralaterally. Right L3 responded differentially to stimuli that were presented ipsi- or contralaterally. Hatching denotes areas responding differentially to contralateral, dots to ipsilateral stimuli.

The triple interaction Vocalization x Valence x Lateralization was significant in A1 and R in the left hemisphere and in A1, R and L3 in the right hemisphere. In *left A1* the significant time window was 22–26 s post-stimulus onset. During this time window the triple interaction was driven by two double interactions (Table 2 and Figure 3). First, the interaction Category x Valence was significant for stimuli presented on the left (but not right or at the center). Second, the interaction Category x Lateralization was significant for positive (but not neutral or negative) stimuli. These interactions were driven by the significant main effect of Category for positive stimuli presented on the left, vocalizations yielding stronger activation than non-vocalizations. *Post-hoc* comparisons revealed during the same time window that among the vocalizations presented on the left positive ones yielded significantly greater activation than neutral or negative ones. Thus, taken together these results highlight in left A1 the pro-eminence of positive vocalizations when presented on the left, i.e., ipsilaterally.

**Table 2:** Differential processing of emotional sounds as function of their category, valence and spatial origin.

Subgroup	Two-way ANOVA			One-way ANOVA	*t*- test
	Category x Valence	Category x Space	Valence x Space	O > P, N	HV > NV
Left stimuli	LH-A1 LH-R RH-R RH-L3	–	–	–	–
Right stimuli	RH-L3	–	–	–	–
Positive stimuli	–	LH-R RH-A1 RH-R RH-L3	–	–	–
Human vocalizations	–	–	RH-L3	–	–
Non-vocalizations	–	–	RH-L3	–	–
Left positive stimuli	–	–	–	–	LH-A1 LH-R RH-R RH-L3
Right positive stimuli	–	–	–	–	RH-L3
Left non-vocalizations	–	–	–	RH-L3	–

In areas, which yielded a significant interaction Category x Valence x Space (i.e., A1 and R bilaterally and L3 on the right side), *post-hoc* analysis were carried out during the relevant timeframes. Subgroups of stimuli (left column) were analyzed with two-way ANOVA Category x Valence, Vocalization x Space, and Valence x Space; one-way ANOVA Valence; as well as with *t*-tests. Early-stage auditory areas yielding significant effects are indicated here. HV, human vocalizations; LH, left hemisphere; N, negative valence; NV, non-vocalizations; O, neutral valence; P, positive valence; RH, right hemisphere.

In *left R* the significant time window for the triple interaction Category x Valence x Lateralization was 18–26 s post-stimulus onset. During this time window the triple interaction was driven by two double interactions (Table 2 and Figure 3). First, the interaction Category x Valence was significant for stimuli presented on the left (but not right or at the center). Second, the interaction Category x Lateralization was significant for positive (but not neutral or negative) stimuli. These two interactions were driven by the significant main effect of Category for positive stimuli presented on the left, vocalizations yielding stronger activation than non-vocalizations. *Post-hoc* comparisons revealed during the same time window that among the vocalizations presented on the left positive ones yielded significantly greater activation than neutral or negative ones. Also positive vocalizations yielded significantly stronger activation when presented on the left than at the center or on the right. Thus, taken together these results highlight in the left R the pro-eminence of positive vocalizations when presented on the left, i.e., ipsilaterally.

In *right A1* the significant time window for the triple interaction Category x Valence x Lateralization was 20–28 s post-stimulus onset. During this time window the triple interaction was driven by two double interactions (Table 2 and Figure 3). First, the interaction Category x Valence was significant for stimuli presented on the left (but not right or at the center). Second, the interaction Category x Lateralization was significant for positive (but not neutral or negative) stimuli. *Post-hoc* comparisons revealed during the same time window that among the vocalizations presented on the left positive ones yielded significantly greater activation than neutral or negative ones. Also positive vocalizations yielded significantly stronger activation when presented on the left than at the center or on the right. Thus, taken together these results highlight in the right A1 the pro-eminence of positive vocalizations when presented on the left, i.e., contralaterally.

In *right R* the significant time window for the triple interaction Category x Valence x Lateralization was 20–28 s post-stimulus onset. During this time window the triple interaction was driven by two double interactions (Table 2 and Figure 3). First, the interaction Category x Valence was significant for stimuli presented on the left (but not right or at the center). Second, the interaction Category x Lateralization was significant for positive (but not neutral or negative) stimuli. These interactions were driven by the significant main effect of Category for positive stimuli presented on the left, vocalizations yielding stronger activation than non-vocalizations. *Post-hoc* comparisons revealed during the same time window that among the vocalizations presented on the left positive ones yielded significantly greater activation than neutral or negative ones. Also positive vocalizations yielded significantly stronger activation when presented on the left than at the center or on the right. Thus, taken together these results highlight in right R the pro-eminence of positive vocalizations when presented on the left, i.e., contralaterally.

In *right L3* the significant time window for the triple interaction Category x Valence x Lateralization was 20–28 s post-stimulus onset. During this time window the triple interaction was driven by three double interactions (Table 2 and Figure 3). First, the interaction Category x Valence was significant for stimuli presented on the left and on the right (but not at the center). The latter was driven by a significant main effect of Category on positive stimuli presented on the right, vocalizations yielding stronger activation than non-vocalizations. Second, the interaction Category x Lateralization was significant for positive (but not neutral or negative) stimuli, driven by a significant main effect of Category on positive stimuli presented on the right or left (but not at the center), vocalizations yielding stronger responses than non-vocalizations. Third, the interaction Valence x Lateralization was significant for vocalizations and for non-vocalizations. The latter was driven by a significant effect of Valence on non-vocalizations presented on the left; neutral non-vocalizations tended to yield stronger responses than positive or negative ones. *Post-hoc* comparisons revealed during the same time window that among the vocalizations presented on the left positive ones yielded significantly greater activation than negative ones. The same was the case among the vocalizations presented on the right, where positive ones yielded significantly greater activation than negative ones. Thus, taken together these results highlight in right L3 the pro-eminence of positive vocalizations when presented on the left or on the right, i.e., contra- or ipsilaterally.

In summary, the results of the triple interaction and of the ensuing double interactions and main effects as well as the *post-hoc* comparisons highlight a significant pre-eminence of the left auditory space for the encoding of positive vocalizations in A1 and R bilaterally. In addition, left and right, but not central space is favored for positive vocalizations in right L3.

The encoding of emotional valence is modulated by the spatial origin of the sound (hypothesis 2).

The interaction Valence x Lateralization was significant bilaterally in VA. In the left hemisphere the significant time window was 10–14 s post-stimulus onset (Figure 1A); *post-hoc* analysis did not yield any significant main effect of Valence at any position nor main effect of Lateralization on any valence (Table 3). In the right hemisphere the interaction Valence x Lateralization was significant during 8–14 s plus 24–28 s (Figure 2A). *Post-hoc* comparison showed that during the latter time window the main effect of valence was significant for sounds presented on the left side (Table 3). In summary, the spatial origin of the sound modulates the encoding of emotional valence within VA.

**Table 3:** Summary of significant double interaction Valence x Lateralization and the ensuing main effects in VA of the left and right hemispheres.

ROI with significant double interaction Valence x Lateralization (time window of significance)	Significant related main effect during the same time window
*Left hemisphere*	
VA (10–14 s)	None
*Right hemisphere*	
VA (8–14 s)	None
VA (24–28 s)	Valence for sounds on left (positive > negative)

For the time window of significant double interaction are listed the related main effects.

The spatial origin of the sound does not appear to impact differently the encoding of vocalizations vs. non-vocalizations (hypothesis 3).

The interaction Category x Lateralization did not yield any significant results in either hemisphere (Figures 1A, 2A).

### Spatial selectivity

A significant main effect of Lateralization was present in the left hemisphere in A1 (during the 10–14 s and 22–26 s time periods); in R (10–14 s and 18–36 s); and in M1 (10–14 s; Figure 1A). The effect was driven by greater activation for contra- than ipsilateral stimuli (Figure 1B).

### Emotional valence modulates the encoding of vocalizations

Significant interaction of Category x Valence was present in either hemisphere. In the left hemisphere this was the case in A1 (12–26 s); R (14–18 s); L1 (10–18 s and 22–26 s); L2 (12–20 s and 24–28 s); M2 (22–26 s); and VA (4–16 s and 20–28 s; Figure 1A). In the right hemisphere this was the case in A1 (14–18 s); R (12–28 s); L1 (14–24 s); L2 (10–26 s); L3 (12–18 s); L4 (14–18 s); M1 (14–18 s and 22–26 s); M3 (30–36 s); M4 (32–36 s); and VA (8–26 s; Figure 2A). In A1, R, L1 and L2 the interactions appeared to be driven by the predominance of positive vocalizations and/or neutral non-vocalizations (Figures 1B, 2B).

A significant main effect of Valence was present in several areas of either hemisphere. In the left hemisphere this was the case A1 (18–30 s); R (20–36 s); L1 (24–36 s); L2 (12–14 s and 18–36 s); L3 (6–12 s and 16–36 s); M1 (16–24 s and 28–36 s); M2 (28–36 s); M4 (16–24 s and 28–36 s); and VA (6–28 s; Figure 1A). In the right hemisphere it was the case in A1 (20–24 s); R (24–36 s); L2 (28–32 s); L3 (6–12 s and 24–36 s); M1 (20–24 s); M2 (18–22 s); M4 (16–24 s and 28–36 s); and VA (8–20 s; Figure 2A). The effect tended to be driven by greater activation by vocalizations with positive rather than negative or neutral valence and by non-vocalizations with neutral rather than positive valence (Figures 1B, 2B).

A significant main effect of Category was present in either hemisphere. In the left hemisphere this was the case in L1 (6–22 s); L2 (6–26 s and 32–36 s); L3 (6–28 s and 32–36 s); M1 (8–12 s); and VA (6–28 s and 32–36 s; Figure 1A). In the right hemisphere this was the case in L2 (6–28 s); L3 (6–26 s); and VA (6–28 s and 32–36 s; Figure 2A). The effect was driven by greater activation by vocalizations than non-vocalizations by overall greater activation by vocalizations than non-vocalizations (Figures 1B, 2B).

## Discussion

Our results indicate that auditory spatial cues modulate the encoding of emotional valence in several early-stage auditory areas and in VA. The most striking effect is the pre-eminence of the left auditory space for the encoding of positive vocalizations. Furthermore, spatial cues appear to render emotional vocalizations more salient, as indicated by comparing our results with those of a previous study (Grisendi et al., 2019). The interactions of the category (human vocalizations vs. other environmental sounds), emotional valence and the spatial origin of the sound characterize the vocalization pathway within the early stage auditory areas and VA.

### Pre-eminence of the left auditory space for positive vocalizations – hemispheric asymmetries

Auditory stimuli presented within the left space elicit stronger responses in A1 and R of the left and right hemisphere when positive vocalizations are used (Figure 3). In both hemispheres neural activity elicited by positive vocalizations presented on the left was higher than neural activity elicited by (i) neutral or negative vocalizations presented at any of the three positions; or (ii) non-vocalizations of any valence at any of the three positions. The involvement of left A1 and R in favor of the ipsilateral and that of right A1 and R in favor of the contralateral, left space speaks against a mere effect of contralateral space or a classical hemispheric dominance.

The stronger encoding of positive vocalizations presented on the left side suggests that they may be more salient than when presented at other positions. The pre-eminence of the left auditory space, which we describe here, is reminiscent of the left-ear advantage, which was reported for emotional dichotic listening tasks in two studies (Erhan et al., 1998; Jäncke et al., 2001). Both studies compared emotional vs. neutral vocalizations, but did not discriminate between positive and negative valence. Their results have been interpreted in terms of right hemispheric competence for emotional processing (see also Gadea et al., 2011). Another series of studies used emotional valence of spoken words for spatial orienting of attention. Emotional word cues presented on the right side introduced spatial attentional bias for the following neutral sound (beep; Bertels et al., 2010). The interpretation of these results was influenced by the assumption that (i) one-sided presentation of auditory stimuli is preferentially treated by the contralateral hemisphere and (ii) the nature of the stimuli – verbal vs. emotional – tends to activate one hemisphere. Thus, the right side bias introduced by emotional words was eventually interpreted as prevailing influence of verbal content (Bertels et al., 2010). The nature of stimuli used in these studies, all verbal vocalizations, and the fact that they were presented mono-aurally, and not lateralized with interaural time (as here) or intensity differences, precludes their interpretation in terms of the emotional value of space.

The left-space preference, which we observed bilaterally in A1 and R, is greater for positive vocalizations than other stimuli. The phenomenon we describe here, the pre-eminent encoding of emotional vocalizations when presented in the left space in left and right R and A1, differs from previously described principles of auditory encoding. First, our results cannot be simply interpreted in terms of the well documented preference of the early-stage auditory areas for the contralateral space. This has been demonstrated for auditory stimuli in general (Deouell et al., 2007; Da Costa et al., 2015; Stecker et al., 2015; McLaughlin et al., 2016; Derey et al., 2017; Higgins et al., 2017) and more recently for auditory stimuli with positive emotional valence, which yielded strong contralateral activity when presented on the left side (Kryklywy et al., 2013). Second, our results do not show lateralization for a given type of stimuli, i.e., a preferential encoding within the left or the right auditory cortex, such as shown for stimuli with rapid formant transition in left auditory cortex (Charest et al., 2009); for varying rates of stimuli in the left and increasing spectral information in the right auditory cortex (Warrier et al., 2009); or more generally the asymmetry of the auditory regions in terms of temporal selectivity (Nourski and Brugge, 2011).

We did not investigate in this first study, whether the pre-eminent encoding of positive vocalizations when presented on the left side differs between male and female subjects, as do parts of the networks controlling speech production (de Lima Xavier et al., 2019).

Further experiments need to clarify whether the preference of R and A1 for positive vocalizations when presented in the left space can be modulated by context and/or attention. The sequence in which auditory stimuli are presented was shown to influence their encoding; the auditory cortex was shown to respond more strongly to pulsed noise stimuli when they are presented to the contra- than ipsilateral ear; this contralateral advantage is no longer present when the same type of monoaural stimuli is interspersed with binaural moving stimuli (Schönwiesner et al., 2007). The right ear advantage in dichotic listening tasks decreases when attention is oriented toward the left ear; this change in performance was shown to be accompanied with decreases in neural activity demonstrated by fMRI (Kompus et al., 2012) and with MEG recordings (Alho et al., 2012).

Although compatible with evidence from previous studies, our results give a different picture of the emotional auditory space and its encoding within the early-stage auditory areas. We have documented a genuine pro-eminence of the left space for positive vocalizations and not simply a right hemispheric or contralateral dominance, the key observation being that left-sided positive vocalizations stand out within the primary auditory cortex of both hemispheres. Several aspects need to be investigated in future studies. There is no current evidence on the behavioral relevance of the emotional pro-eminence of the left auditory space. It is unclear when it emerges in human development; indirect evidence comes from studies that reported left-ear preference for emotional sounds in children (Saxby and Bryden, 1984; Obrzut et al., 2001). The emotional pro-eminence of the left auditory space may not be an exclusively human characteristic. Although not explored as such in non-human primates, the reported right-hemispheric dominance for the processing of emotional sounds may be a correlate of the emotional pro-eminence of the left auditory space [for review (Gainotti, 2022)].

### Spatial cues make emotional vocalizations more salient

**Figure 4:**
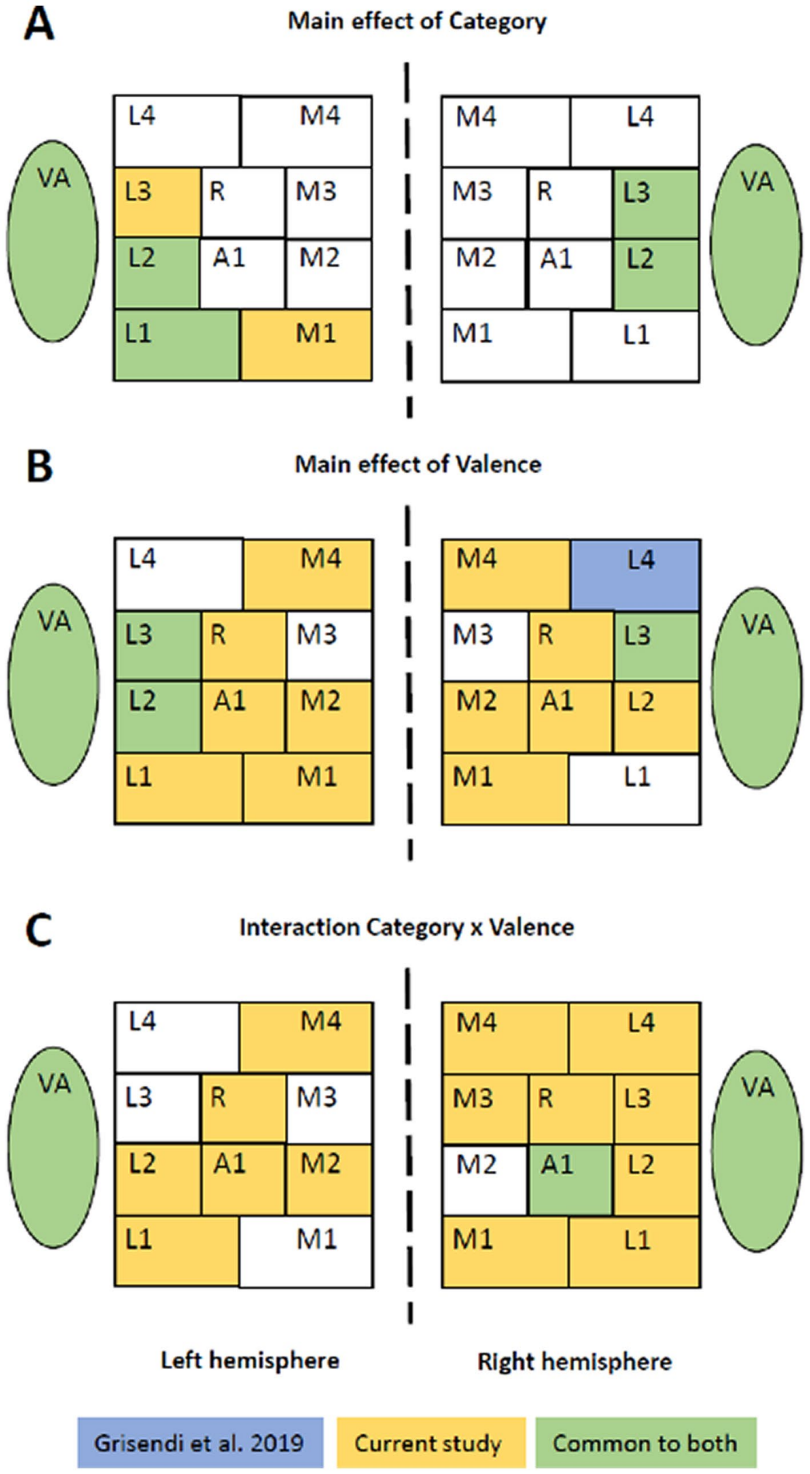
Emotional sounds with or without spatial cues. Juxtaposition of the results from the two-way and three-way ANOVAs found in the present and a previous study (Grisendi et al., 2019), which used the same set of stimuli, the same paradigm and an ANOVA based statistical approach. The former used lateralized stimuli, whereas the latter did not. Whereas the main effect of Category highlights in both studies a very similar set of areas **(A)**, the main effect of Valence **(B)** and the interaction Category x Valence **(C)** revealed significant differences in more areas when stimuli were lateralized.

Two of our observations suggest that spatial cues render emotional vocalizations more salient. First, positive vocalizations presented on the right or the left were prominent in right L3 (Table 2). Second, the use of spatial cues appeared to enhance the salience of emotional valence in several early-stage areas. In a previous study, the same set of stimuli (human vocalizations and non-vocalizations of positive, neutral and negative valence), the same paradigm and an ANOVA based statistical analysis were used, albeit without lateralization (Grisendi et al., 2019). The juxtaposition of the distribution of significant interactions and significant main effects in early-stage areas and in VA highlights striking differences, which concern almost exclusively the factor Valence (and not Category; Figure 4). Main effect of Category highlighted in both studies a very similar set of areas, with vocalizations yielding greater activation than non-vocalizations. Main effect of Valence was strikingly dissimilar, being significant in many more areas when spatial cues were used. The same was observed for the interaction Category x Valence, with many more areas being significant when spatial cues were used; it is to be noted that in both studies the interaction was driven by greater responses to positive vocalizations. This increased saliency when spatial cues are used is not due to a modulation of emotional valence by lateralization; this interaction was only significant in VA but not in any of the early-stage areas.

The mechanisms by which spatial cues confer greater salience to emotional vocalizations is currently unknown. Interaural interactions during first cortical processing stages may enhance emotional stimuli, as does increasing intensity (Bach et al., 2008, 2009). Further studies are needed to investigate whether the effect is associated uniquely with interaural time differences (used here) or whether interaural intensity differences or more complex spatial cues have the same effect.

### Voice area: vocalizations are selectively modulated by emotional valence but not spatial cues

Our analysis clearly showed that within VA the encoding of vocalizations is modulated by emotional valence, as did a series of previous studies (Belin et al., 2002; Grandjean et al., 2005; Ethofer et al., 2006, 2008, 2009, 2012; Beaucousin et al., 2007; Obleser et al., 2007, 2008; Bestelmeyer et al., 2017; Grisendi et al., 2019). The new finding is that this clear modulation of vocalizations by emotional valence is not paralleled by a modulation by the spatial origin of the sound. This is reminiscent of the findings of Kryklywy et al. (2013), who reported that emotional valence, but not spatial attributes, impacts the processing within the ventral stream on the temporal convexity. Their stimuli consisted to 75% of human vocalizations and may have driven the effect they observed.

In our study spatial information did not modulate significantly the encoding of vocalizations within VA. However, the spatial origin impacted the activity elicited by sound objects in general. Thus, positive and neutral sounds; i.e., vocalizations and non-vocalizations taken together, yielded stronger response than negative ones when presented on the left or on the right, as compared to a presentation at the center. This preference for positive and neutral sounds when presented in lateral space was present in both hemispheres.

## Conclusion

Previous behavioral studies (Erhan et al., 1998; Jäncke et al., 2001; Bertels et al., 2010) indicated that spatial origin impacts emotional processing of sounds, possibly via a preferential encoding of the contralateral space on the supratemporal plane (Kryklywy et al., 2013, 2018). We demonstrate here that there is a preference in terms of space, and not hemisphere, with a clear pre-eminence of the left auditory space for positive vocalizations. Positive vocalizations presented on the left side yield greater activity in bilateral A1 and R. VA does not share the same preference for the left space. Comparison with a previous study (Grisendi et al., 2019) indicates that spatial cues may render emotional valence more salient within the early-stage auditory areas.

## Data availability statement

The raw data supporting the conclusions of this article will be made available by the authors, without undue reservation.

## Ethics statement

The studies involving human participants were reviewed and approved by the Ethical Committee of the Canton de Vaud (reference number 282/08). The patients/participants provided their written informed consent to participate in this study.

## Author contributions

TG, SC, and SD contributed to the elaboration of the experimental design, the interpretation of the data, and the manuscript preparation. TG and SD contributed to the recruitment of the participants, the data acquisition and analysis. All authors approved the actual version of the manuscript.

## Funding

This work was supported by the Swiss National Science Foundation Grant to SC (FNS 320030-159708) and by the Centre d’Imagerie BioMédicale (CIBM) of the UNIL, UNIGE, HUG, CHUV, EPFL and the Leenaards and Jeantet Foundations.

## Conflict of interest

The authors declare that the research was conducted in the absence of any commercial or financial relationships that could be construed as a potential conflict of interest.

## Publisher’s note

All claims expressed in this article are solely those of the authors and do not necessarily represent those of their affiliated organizations, or those of the publisher, the editors and the reviewers. Any product that may be evaluated in this article, or claim that may be made by its manufacturer, is not guaranteed or endorsed by the publisher.

## Footnotes

^1^ www.psychtoolbox.org

^2^ https://audacityteam.org

^3^ http://www.fon.hum.uva.nl/praat/

## Abbreviations

A1: primary auditory area
HVN: human vocalizations with negative emotional valence
HVP: human vocalizations with positive emotional valence
HV0: human vocalizations with neutral emotional valence
NVN: non-vocalizations with negative emotional valence
NVP: non-vocalizations with positive emotional valence
NV0: non-vocalizations with neutral emotional valence
R: rostral (primary) auditory area
VA: voice area
